# Recreational Water–Associated Disease Outbreaks — United States, 2009–2010

**Published:** 2014-01-10

**Authors:** Michele C. Hlavsa, Virginia A. Roberts, Amy M. Kahler, Elizabeth D. Hilborn, Timothy J. Wade, Lorraine C. Backer, Jonathan S. Yoder

**Affiliations:** 1 Div of Foodborne, Waterborne, and Environmental Diseases, National Center for Emerging and Zoonotic Infectious Diseases, CDC; 2 Environmental Protection Agency; 3 Div of Environmental Hazards and Health Effects, National Center for Environmental Health, CDC

Recreational water–associated disease outbreaks result from exposure to infectious pathogens or chemical agents in treated recreational water venues (e.g., pools and hot tubs or spas) or untreated recreational water venues (e.g., lakes and oceans). For 2009–2010, the most recent years for which finalized data are available, public health officials from 28 states and Puerto Rico electronically reported 81 recreational water–associated disease outbreaks to CDC’s Waterborne Disease and Outbreak Surveillance System (WBDOSS) via the National Outbreak Reporting System (NORS). This report summarizes the characteristics of those outbreaks. Among the 57 outbreaks associated with treated recreational water, 24 (42%) were caused by *Cryptosporidium*. Among the 24 outbreaks associated with untreated recreational water, 11 (46%) were confirmed or suspected to have been caused by cyanobacterial toxins. In total, the 81 outbreaks resulted in at least 1,326 cases of illness and 62 hospitalizations; no deaths were reported. Laboratory and environmental data, in addition to epidemiologic data, can be used to direct and optimize the prevention and control of recreational water–associated disease outbreaks.

CDC defines a recreational water–associated disease outbreak as the occurrence of similar illnesses in two or more persons epidemiologically linked by location and time of exposure to recreational water or water-associated chemicals volatilized into the air surrounding the water. Public health officials in U.S. states, the District of Columbia, U.S. territories, and Freely Associated States* voluntarily report outbreaks of recreational water–associated illness to CDC. This report summarizes data on recreational water–associated disease outbreaks electronically reported to CDC’s WBDOSS via NORS† by October 3, 2012, in which the earliest illness onset date occurred during 2009–2010. Data requested for each outbreak include the number of cases,§ hospitalizations, and deaths; illness type; etiology; the venue (e.g., hot tub or spa) and setting (e.g., hotel) at which the outbreak exposure occurred; and earliest illness onset date. Additionally, 10 states received CDC funding to conduct enhanced surveillance for human and animal illnesses and deaths associated with exposure to cyanobacterial toxins¶; outbreaks identified by these surveillance efforts were also voluntarily reported to NORS. Negative binomial regression analyses were conducted to assess for trends in incidence. All outbreaks were classified according to the strength of data implicating recreational water as the outbreak vehicle, as described elsewhere (1).** Classification does not necessarily assess adequacy or completeness of investigations.††

For 2009–2010, public health officials from 28 states and Puerto Rico reported 81 recreational water–associated disease outbreaks (http://www.cdc.gov/healthywater/surveillance/recreational/tables.html) (Figure 1). The number of outbreaks reported for a given year (range: 6–84 outbreaks) has significantly increased (p<0.001) since 1978, the year national reporting of recreational water–associated disease outbreaks began (Figure 2). The 1,326 outbreak-related cases reported for 2009–2010 resulted in at least 62 (5%) hospitalizations; no outbreak-related deaths were reported. Etiology was confirmed for 49 (60%) outbreaks, of which 27 (55%) were caused by *Cryptosporidium* (Table). Since 1988, the year the first U.S. treated recreational water–associated outbreak of cryptosporidiosis was detected, the number of these outbreaks reported for a given year (range: 0–40 outbreaks) has significantly increased (p<0.001) and has, at least in part, driven the significant increase in the overall number of recreational water–associated disease outbreaks reported for a given year (p<0.001) (Figure 2). Based on data reported to CDC, 32 (40%) of the 81 outbreaks were categorized as class IV, “epidemiologic and clinical laboratory data provided but limited, and environmental data not provided or inadequate.”

Of the 81 outbreaks during 2009–2010, 57 (70%) were associated with treated recreational water. These outbreaks resulted in at least 1,030 cases (78% of all outbreak-related cases) and 40 (65%) hospitalizations. The median number of cases reported for these outbreaks was seven (range: 2–280 cases). The outbreaks had a bimodal temporal distribution (Figure 1). Of the 25 (44%) outbreaks that started in July or August, 23 (92%) were of acute gastrointestinal illness, and 21 (84%) were caused by *Cryptosporidium*. Ten (18%) outbreaks started in March; five (50%) were of an unidentified etiology but were suspected to have been caused by pool chemicals or disinfection by-products. Over half of the 57 outbreaks were associated with hotel (19 [33%]) or waterpark (14 [25%]) settings. Outbreaks associated with the hotel setting most frequently started in February, March, or April (11 [58%]); were outbreaks of dermatologic illnesses, conditions, or symptoms confirmed or suspected to have been caused by *Pseudomonas aeruginosa* (nine [47%]); and were epidemiologically linked, at least in part, to a hot tub or spa (11 [58%]).

Of the 81 outbreaks during 2009–2010, 24 (30%) were associated with untreated recreational water. These outbreaks resulted in at least 296 cases (22% of all outbreak-related cases) and 22 (35%) hospitalizations. The median number of cases reported for these outbreaks was 5.5 (range: 2–69 cases). Of these outbreaks, 23 (96%) were associated with fresh water; 20 (83%) started in June, July, or August; and 11 (46%) were confirmed or suspected to have been caused by cyanobacterial toxins. A more detailed description of data on outbreaks at least suspected to have been caused by cyanobacterial toxins is separately presented in this issue of *MMWR*.

## Editorial Note

The reporting of all waterborne disease outbreaks to CDC transitioned from paper-based to electronic, starting in 2009. The 2009–2010 counts of 81 outbreaks and 1,326 total cases are less than the 134 outbreaks and 13,966 cases (55% of which were associated with two communitywide outbreaks) (1–3), reported for 2007–2008. Thus, the 2009–2010 data represent a 40% and 91% decrease in the number of reported outbreaks and outbreak-related cases, respectively. The number of outbreaks reported for 2007–2008 might be an outlier because a possible 2007 multistate treated recreational water–associated outbreak of cryptosporidiosis was counted as multiple separate outbreaks in adjacent states (1). With no more than 126 cases reported for any given 2009–2010 recreational water–associated outbreak of cryptosporidiosis, perhaps state and local public health officials are proactively instituting prevention and control measures and thus preventing or minimizing communitywide cryptosporidiosis outbreaks (4). However, without systematic molecular typing of *Cryptosporidium* isolates, it is difficult to determine if identified outbreaks are independent or related events.

The Model Aquatic Health Code (MAHC) is a set of science-based and best-practice guidelines designed to reduce risk for illness and injury at public treated recreational–water venues (http://www.cdc.gov/mahc). Thus, the MAHC represents an opportunity to prevent and control outbreaks through recommendations such as additional water treatment (e.g., ultraviolet light or ozone) to inactivate extremely chlorine-tolerant *Cryptosporidium* oocysts at venues where WBDOSS data indicate there is increased risk for transmission. In the United States, codes regulating public treated recreational–water venues are independently written and enforced by state or local agencies; the consequent variation in the codes has been identified as a barrier to preventing and controlling outbreaks associated with these venues. Since 2007, CDC, the New York State Department of Health, and many other stakeholders have spearheaded the development of the MAHC. The MAHC will be available for the second and final round of public comment in early 2014; the first official edition is expected to be released in the summer of 2014.

Eutrophication§§ of natural waters can potentially lead to harmful algal blooms (HABs), which naturally release cyanobacterial toxins. Illness caused by cyanobacterial toxins is nonspecific and probably underrecognized; thus, its epidemiology is not well understood and warrants further investigation. No U.S. federal regulations or public health guidelines specify allowed concentrations of cyanobacterial toxins in the water; however, some U.S. states have developed their own guidelines (5,6). The economic losses associated with HABs are substantial. A study of the economic impact of *Karenia brevis*, a harmful marine alga, in one Florida county during 2001–2006 estimated the cost of emergency department treatment of bloom-associated respiratory illness to range from $500,000 to $4 million (7). Closing or not using U.S. freshwater lakes for recreational activities because of hypereutrophication (i.e., HABs) is estimated to cost $0.37–1.16 billion per year (8).

The findings in this report are subject to at least two limitations. First, the outbreak counts presented are likely an underestimate of actual incidence. Factors such as 1) limited illness severity, 2) small outbreak size, 3) long incubation period of illness, 4) wide geographic dispersion of ill swimmers, 5) transient nature of contamination, 6) setting or venue of outbreak exposure (e.g., residential backyard pool), and 7) potential lack of communication between those who respond to outbreaks of chemical etiology (e.g., hazardous materials personnel) and those who usually report outbreaks (e.g., infectious disease epidemiologists) can be barriers to the detection, investigation, and reporting of outbreaks. Second, the jurisdictions reporting outbreaks most frequently might not be the jurisdictions in which the outbreaks most frequently occur, because public health capacity and notification requirements for diseases and outbreaks vary across jurisdictions.

The transition from paper-based to electronic reporting of national waterborne disease outbreak surveillance data represents an opportunity to optimize the quality and completeness of epidemiologic, clinical laboratory, and environmental data reported for individual outbreaks. CDC is working to 1) collaboratively identify, with state partners, NORS reporting issues and address them, 2) expand systematic molecular typing of *Cryptosporidium* isolates nationally (http://www.cdc.gov/parasites/crypto/cryptonet.html), 3) develop tools to collect environmental data during inspections of venues implicated in outbreak investigations, and 4) provide funding to state partners to support enhanced surveillance of waterborne disease. Additionally, establishing a national surveillance system that collects state and local environmental data on routine inspection of public treated recreational–water venues can be used to assess code compliance and highlight where violations are disproportionately high (9). Combining these data and data from systematic molecular typing of *Cryptosporidium* isolates with NORS data could synergistically direct and optimize prevention and control of recreational water–associated disease outbreaks.

What is already known on this topic?

Recreational water–associated disease outbreaks continue to occur throughout the United States. CDC collects data on waterborne disease outbreaks electronically submitted by states, the District of Columbia, U.S. territories, and Freely Associated States to CDC’s Waterborne Disease and Outbreak Surveillance System via the National Outbreak Reporting System.

What is added by this report?

For 2009–2010, a total of 81 recreational water–associated disease outbreaks were reported to CDC. Of the 1,326 reported outbreak-related cases, 62 resulted in hospitalization; no deaths were reported. Almost a third (30%) of the outbreaks were caused by *Cryptosporidium* and associated with treated recreational water venues (e.g., pools). Of 24 outbreaks associated with untreated recreational water venues (e.g., lakes), almost half (46%) were confirmed or suspected to have been caused by cyanobacterial toxins.

What are the implications for public health practice?

Guidance to prevent and control recreational water–associated disease outbreaks, such as the Model Aquatic Health Code, can be optimized when directed by national outbreak data as well as laboratory data (e.g., molecular typing of *Cryptosporidium*) and environmental data (i.e., inspection data).

## Figures and Tables

**FIGURE 1:**
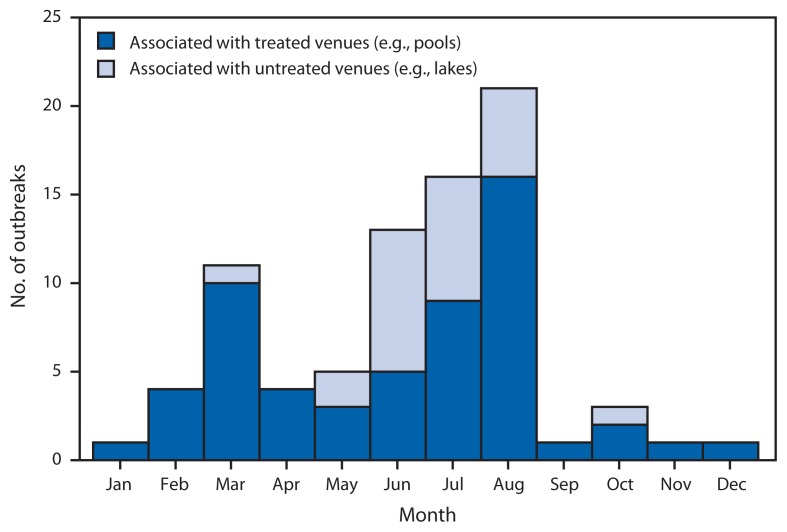
Number of waterborne disease outbreaks associated with recreational water (n = 81), by month — United States, 2009–2010 **Source:** CDC’s Waterborne Disease and Outbreak Surveillance System, as reported via the National Outbreak Reporting System.

**FIGURE 2:**
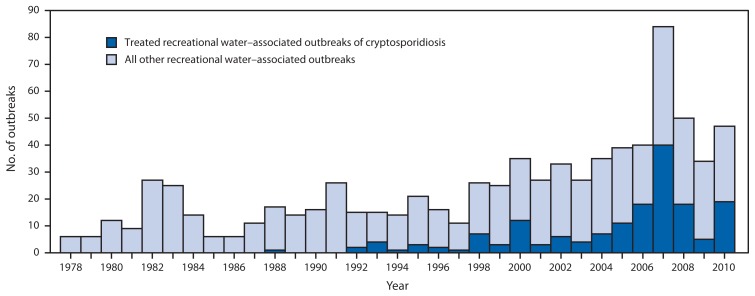
Number of waterborne disease outbreaks associated with recreational water (n = 789), by year — United States, 1978–2010 **Source:** CDC’s Waterborne Disease and Outbreak Surveillance System, as reported via the National Outbreak Reporting System.

**TABLE:** Number of waterborne disease outbreaks associated with recreational water (n = 81), by etiology and type of water exposure — United States, 2009–2010

Etiology	Type of water exposure						Total		

	Treated			Untreated					
		
	Outbreaks	Cases*	Hospitalized	Outbreaks	Cases*	Hospitalized	Outbreaks (%)†	Cases* (%)	Hospitalized (%)
**Bacterium**	**10**	**75**	**11**	**5**	**91**	**18**	**15 (19)**	**166 (13)**	**29 (47)**
*Campylobacter jejuni*	0	0	0	1	6	4	**1**	**6**	**4**
*Escherichia coli* O157:H7	1	14	4	3	17	8	**4**	**31**	**12**
*Legionella* spp.	4	8	7	0	0	0	**4**	**8**	**7**
*Pseudomonas aeruginosa*	4	50	0	0	0	0	**4**	**50**	**0**
*Shigella sonnei*	1	3	0	1	68	6	**2**	**71**	**6**
**Parasite**	**25**	**413**	**15**	**3**	**16**	**0**	**28 (35)**	**429 (32)**	**15 (24)**
*Cryptosporidium* spp.	24	406	14	3	16	0	**27**	**422**	**14**
*Giardia intestinalis*	1	7	1	0	0	0	**1**	**7**	**1**
**Virus**	**0**	**0**	**0**	**1**	**69**	**2**	**1 (1)**	**69 (5)**	**2 (3)**
Norovirus	0	0	0	1	69	2	**1**	**69**	**2**
**Chemical**	**0**	**0**	**0**	**4**	**38**	**1**	**4 (5)**	**38 (3)**	**1 (2)**
Cyanobacterial toxin(s)§	0	0	0	4	38	1	**4**	**38**	**1**
**Multiple** ¶	**0**	**0**	**0**	**1**	**45**	**0**	**1 (1)**	**45 (3)**	**0 (0)**
*Campylobacter jejuni*, norovirus genogroup I, *Shigella* sp.	0	0	0	1	45	0	**1**	**45**	**0**
**Unidentified**	**22**	**542**	**14**	**10**	**37**	**1**	**32 (40)**	**579 (44)**	**15 (24)**
Suspected avian schistosomes	0	0	0	2	11	0	**2**	**11**	**0**
Suspected chemical exposure**	8	54	1	0	0	0	**8**	**54**	**1**
Suspected chloramines**	2	311	0	0	0	0	**2**	**311**	**0**
Suspected algaecide (copper)	0	0	0	1	3	0	**1**	**3**	**0**
Suspected cyanobacterial toxin(s)§	0	0	0	7	23	1	**7**	**23**	**1**
Suspected norovirus	2	91	13	0	0	0	**2**	**91**	**13**
Suspected *P. aeruginosa*	5	55	0	0	0	0	**5**	**55**	**0**
Unidentified	5	31	0	0	0	0	**5**	**31**	**0**
**Total (%)**	**57 (70)**	**1,030 (78)**	**40 (65)**	**24 (30)**	**296 (22)**	**22 (35)**	**81 (100)**	**1,326 (100)**	**62 (100)**

**Source:** CDC’s Waterborne Disease and Outbreak Surveillance System, as reported via the National Outbreak Reporting System.

* No deaths were reported among cases associated with these outbreaks.

† Percentages do not add up to 100% because of rounding.

§ Confirmed or suspected cyanobacterial toxin etiologies were determined on the basis of symptom and environmental data. Microcystin was considered a confirmed etiology if water testing detected ≥20 parts *μ*g/mL microcystin toxin in water samples collected during or within 1 day of the outbreak exposure period. Microcystin was considered a suspected etiology if water testing detected <20 *μ*g/mL microcystin toxin in water samples collected during or within 1 day of the outbreak exposure period. All other algal toxins (e.g., saxitoxin) measured in water samples collected during or within 1 day of the outbreak exposure period were considered suspected etiologies, regardless of the toxin level. A general etiology of “cyanobacterial toxin(s)” was considered to be a suspected etiology if environmental data were insufficient to identify specific toxins or if rash was a predominant illness in an outbreak for which confirmed or suspected etiologies are not well known to cause rash (i.e., to acknowledge the potential for illness caused by undetected mixed algal blooms, exotoxins, or endotoxins).

¶ Outbreaks with multiple etiologies are defined as outbreaks in which more than one type of etiologic agent (e.g., bacterium or virus) is detected in specimens from affected persons. Clinical test results are currently reported at the person level (e.g., five of 10 persons tested positive for *Cryptosporidium*) in the National Outbreak Reporting System. Clinical test results were historically reported to CDC at the clinical specimen level (e.g., five of 10 stool specimens tested positive for *Cryptosporidium*). Multiple etiologies were assigned when each etiologic agent was found in ≥5% of positive clinical specimens. Therefore, multiple etiology assignments presented in this report might not be directly comparable with previously published data.

** Etiology unidentified: disinfection by-products (e.g., chloramines), altered water chemistry, or extremely elevated chlorine levels suspected based on reported data.

## Footnotes

* Includes Marshall Islands, Federated States of Micronesia, and Republic of Palau; formerly part of the U.S.-administered Trust Territory of the Pacific Islands.

† The reporting form and guidance for reporting via NORS are available at http://www.cdc.gov/nors. Disease outbreaks resulting from recreational water exposures on cruise ships are not reported to WBDOSS.

§ For this report, case counts were based on the estimated number of total cases; reporting public health agencies were not asked to provide supporting evidence for estimates as they were for estimates in the previous report (1).

¶ Harmful Algal Bloom-Related Illness Surveillance System activities were supported through the Cooperative Agreement to Enhance Surveillance of Risk Factors and Health Effects Related to Harmful Algal Blooms (CDC-RFA-EH08-801).

** Ranging from class I (provided adequate epidemiologic, clinical laboratory, and environmental data) to class IV (epidemiologic and clinical laboratory data provided but limited, and environmental data not provided or inadequate).

†† Outbreaks and subsequent investigations occur under different circumstances, and not all outbreaks can be investigated vigorously. Multiple factors (e.g., timeliness of outbreak detection) contribute to the ability to collect and report optimal epidemiologic, clinical laboratory, and environmental data.

§§ High concentrations of nutrients stimulate plant growth. Eutrophication is associated with widely varying concentration of dissolved oxygen, algal blooms, and a decline in aquatic animal life.
